# Crystal structure of (18-crown-6)potassium(I) [(1,2,3,4,5-η)-cyclo­hepta­dien­yl][(1,2,3-η)-cyclo­hepta­trien­yl]cobalt(I)

**DOI:** 10.1107/S2056989015003151

**Published:** 2015-02-21

**Authors:** William W. Brennessel, John E. Ellis

**Affiliations:** a Department of Chemistry, 120 Trustee Road, University of Rochester, Rochester, NY 14627, USA; b Department of Chemistry, 207 Pleasant Street SE, University of Minnesota, Minneapolis, MN 55455, USA

**Keywords:** crystal structure, 18-crown-6, hexa­oxa­cyclo­octa­deca­ne, cyclo­hepta­dien­yl, cyclo­hepta­trien­yl, cobalt(I)

## Abstract

The reaction of bis­(anthracene)cobaltate(−I) with excess cyclo­hepta­triene, C_7_H_8_, resulted in a new 18-electron cobaltate containing two different seven-membered ring ligands, based on single-crystal X-ray diffraction. The asymmetric unit of this structure contains two independent cation–anion pairs of the title complex, [K(18-crown-6)][Co(η^3^-C_7_H_7_)(η^5^-C_7_H_9_)], where 18-crown-6 stands for 1,4,7,10,13,16-hexa­oxa­cyclo­octa­decane (C_12_H_24_O_6_), in general positions and well separated. Each (18-crown-6)potassium cation is in contact with the η^3^-coordinating ligand of one cobaltate complex. Each η^3^-coordinating ligand behaves as an allylic anion whose *exo*-diene moiety is bent away from the allylic plane, and thus is not involved directly in the bonding. The metal-coordinating portions of the anionic η^5^ ligands are planar and one of these ligands is modeled as disordered over two positions, with occupancy ratio 0.699 (5):0.301 (5), such that one orientation is rotated by one carbon atom with respect to the other. The diffraction intensities were integrated according to non-merohedral twin law [-1 0 0/0 -1 0/0.064 0 1], a 180° rotation about reciprocal lattice axis [001], and the masses of the twin domains refined to equal amounts. As both ligands are formally coordinated as anions, the cobalt atom is best considered to be Co^I^. This compound is of inter­est as the first to possess cyclo­hepta­trienyl and cyclo­hepta­dienyl ligands in an anionic metal complex.

## Abstract 2

The reaction of bis­(anthracene)cobaltate(−I) with excess cyclo­hepta­triene, C_7_H_8_, resulted in a new 18-electron cobaltate containing two different seven-membered ring ligands, based on single-crystal X-ray diffraction. This compound is of inter­est as the first to possess cyclo­hepta­trienyl and cyclo­hepta­dienyl ligands in an anionic metal complex.

## Chemical context   

To date there is only one crystal structure reported of a homoleptic cyclo­hepta­triene (CHT) transition metal complex, Zr(η^6^-C_7_H_8_)_2_ (Green & Walker, 1989 ▸), presumably because such mol­ecules tend to isomerize. In the case of this zirconium species, room-temperature syntheses produced a mixture of it and its hydrogen-migrated isomer Zr(η^7^-C_7_H_7_)(η^5^-C_7_H_9_). For the titanium analog, although the homoleptic CHT complex was initially observed by NMR, no crystals were obtained, and it readily isomerized. Metal vapor co-condensation reactions of titanium and iron with CHT also led to the isomerized forms (Timms & Turney, 1976 ▸; Blackborow *et al.*, 1976 ▸). Co-condensation of molybdenum atoms with CHT resulted in Mo(η^6^-C_7_H_8_)_2_, which could be isolated at room temperature, but was observed to isomerize to Mo(η^7^-C_7_H_7_)(η^5^-C_7_H_9_) with a half-life of *ca* 200 h (Green *et al.*, 1989 ▸).

Given the tendency for homoleptic CHT complexes to isomerize, we decided to investigate whether this would occur in the late transition metal low-valent cobalt system. The 18-electron anionic precursor bis­(anthracene)cobaltate(−I) was chosen because it had been demonstrated that the anthracene ligands are quite labile (Brennessel *et al.*, 2002 ▸; Brennessel & Ellis, 2012 ▸). Under an argon atmosphere, excess CHT was introduced dropwise to a cold tetra­hydro­furan solution of bis­(anthracene)cobaltate(−I). Red–brown single crystals of the isolated product suitable for an X-ray diffraction experiment revealed a new 18-electron cobalt complex anion containing two different cyclic ligands, [Co(η^3^-C_7_H_7_)(η^5^-C_7_H_9_)]^−^, which confirmed that isomerization had occurred and that both anthracene ligands had been displaced. As no spectroscopy had been performed, it is unknown if an anionic inter­mediate like ‘[Co(η-C_7_H_8_)_2_]^−^’ was initially formed, and if formed, whether it had any lifetime in cold and/or room-temperature solutions.
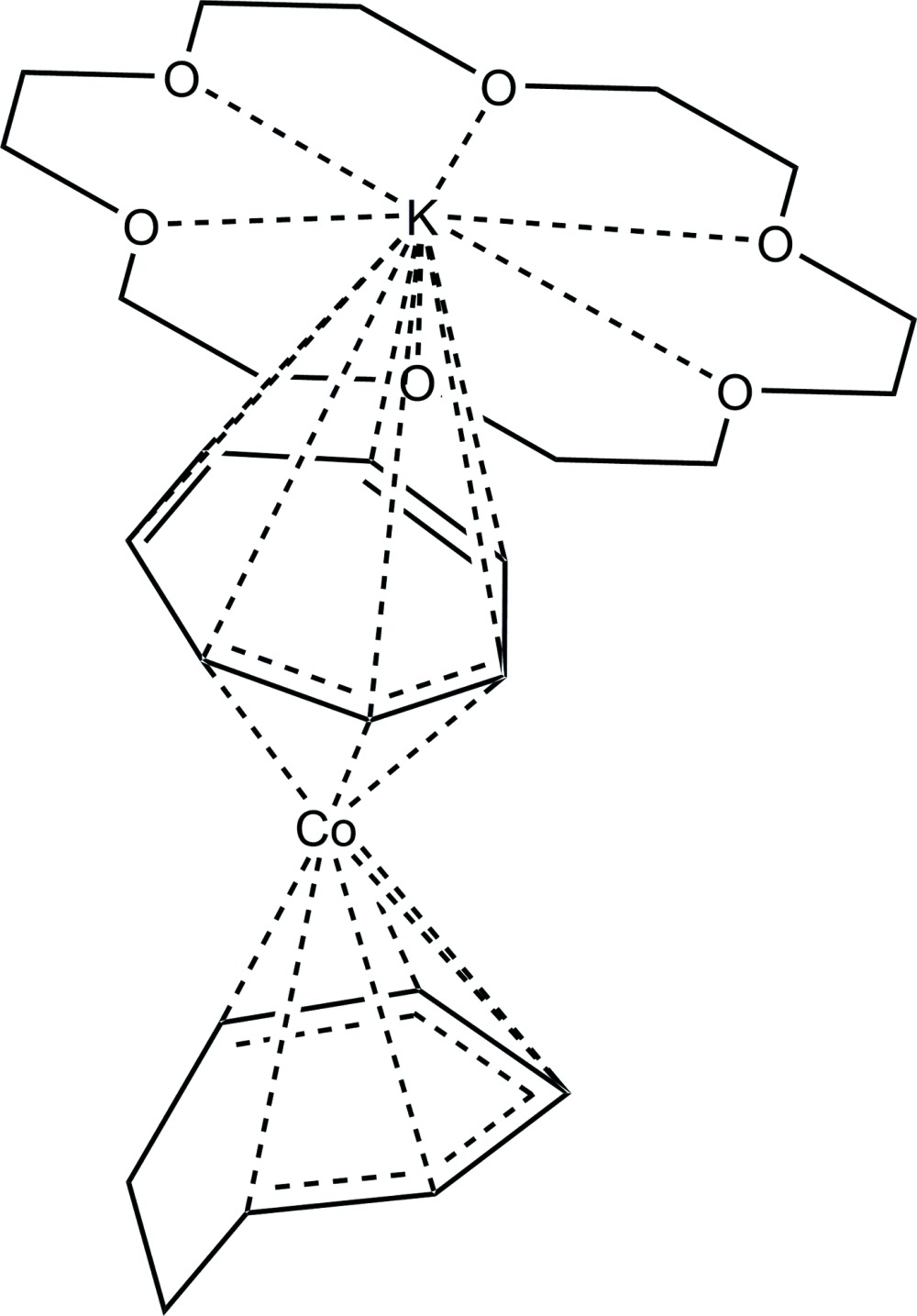


## Structural commentary   

**Figure 1:**
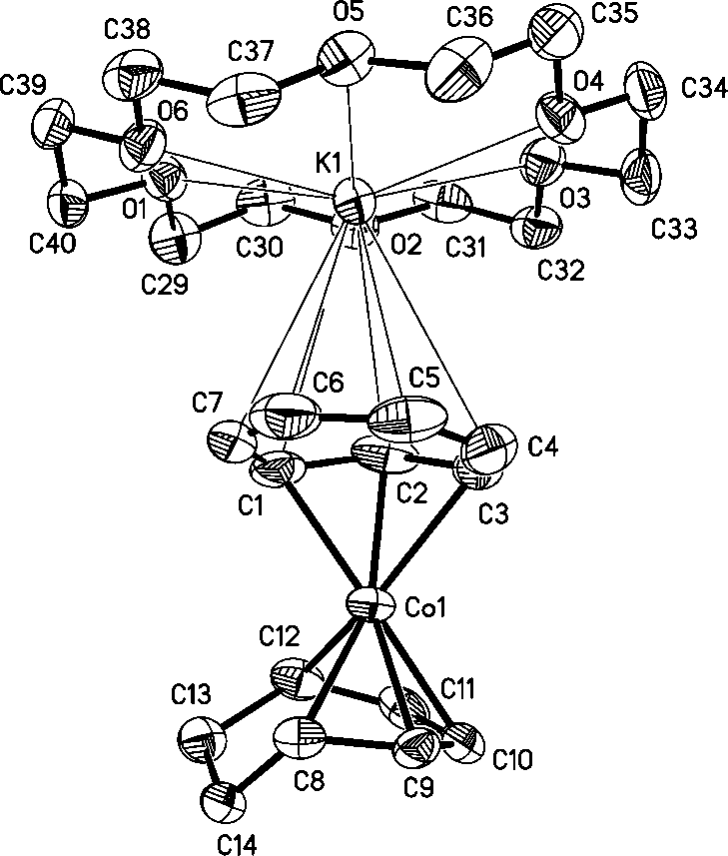
Structure of the first independent mol­ecule of (I), with displacement ellipsoids shown at the 50% probability level. H atoms have been omitted. Thin lines indicate the primarily electrostatic inter­actions between the K^+^ cation and the crown ether and η^3^ ring.

There are two independent contact ion pairs of [K(18-crown-6)][Co(η^3^-C_7_H_7_)(η^5^-C_7_H_9_)], (I), in the asymmetric unit (Figs. 1 ▸ and 2 ▸). The potassium cations are complexed by 18-crown-6 cyclic ethers and are in contact with carbon atoms of the η^3^-coordinating ligands of the cobalt anions, with K⋯C distances ranging from 3.207 (3) to 3.538 (4) Å. The longest K⋯C distance is well within the sum of the van der Waals radii for potassium and carbon of 4.45 Å (Bondi, 1964 ▸). The C_7_H_7_ ligands are bonded η^3^ to the cobalt atoms, and their Co—C and C C bond lengths are consistent with their formulations as anionic allylic ligands with *exo*-diene moieties, *i.e.*, η^3^-cyclo­hepta­trienyl ligands (see Table 1 ▸). Especially noteworthy are the lengths of the double bonds in the *exo*-diene portions, which are normal for C=C bonds and show that the *exo*-diene moieties are independent of the allylic coordination to the metal centers. The Co—C bond lengths have the typical long–short–long pattern seen in other η^3^-cyclo­hepta­trienyl transition metal species (Table 2 ▸ ▸), and the *exo*-diene portions of these ligands are essentially planar and are bent away from the plane of the allylic regions by 28.0 (4) and 27.2 (4)°, for anions containing Co1 and Co2, respectively. Inter­estingly, the tropylium cation (CHT^+^) also has the formula C_7_H_7_; however, tropylium as a ligand is aromatic, and thus planar and with similar C C bond lengths. The η^5^-coordinating ligands are essentially planar in their cobalt-bonded regions with r.m.s. deviations from planarity of 0.050 and 0.051 Å for planes C8–C12 and C22–C26, respectively (see Figs. 1 ▸ and 2 ▸).

With cobalt bound to three (‘all­yl’) and five (‘penta­dien­yl’) carbon atoms of the seven-membered rings as described above, it is thought best to consider the cobalt atom as formally Co^I^ with two anionic ligands. Extended Hückel MO calculations on [Fe(η^3^-C_7_H_7_)(CO)_3_]^−^ (Hofmann, 1978 ▸), whose structure has been reported (Sepp *et al.*, 1978 ▸), not only demonstrated that there is a preference for the metal to bind through the η^3^-allylic region of the ligand rather than through the diene segment, but showed that there is more charge localization on the ring for the former conformation over the latter.

The exact mechanism of isomerization has not been determined for (I), including whether the hydrogen transfer is intra- or inter­molecular. In one DFT study on selected early transition metal complexes, the mechanism for hydrogen migration was determined to be intra­molecular, and a metal hydride inter­mediate was predicted to be favored over a direct ligand-to-ligand transfer (Herbert *et al.*, 2004 ▸). The same conclusion was reached in kinetic studies on similar molybdenum complexes (Green *et al.*, 1989 ▸). If these studies can be extended to the cobalt system, then it could be proposed that the hydrogen migration occurs *via* a ‘[CoH(η-C_7_H_7_)(η-C_7_H_8_)]^−^’ inter­mediate.

## Database survey   

As mentioned above, there is exactly one homoleptic CHT structure in the Cambridge Structural Database to date (CSD, Version 5.36, update No. 1, November 2014; Groom & Allen, 2014 ▸), namely Zr(η^6^-C_7_H_8_)_2_ (Green & Walker, 1989 ▸). All others have been structurally characterized after isomerization, including (I). There are 23 structures containing an η^5^-cyclo­hepta­dienyl ligand, but only 12 structures containing an η^3^-cyclo­hepta­trienyl ligand bonded to a single metal atom. Of the latter, just three are anionic; they are of the form [AsPh_4_][*M*(CO)_3_(η^3^-C_7_H_7_)], *M* = Fe (Sepp *et al.*, 1978 ▸) and *M* = Ru, Os (Astley *et al.*, 1990 ▸). (I) is the first example of an anionic transition metal complex containing both cyclo­hepta­dienyl and cyclo­hepta­trienyl ligands to be reported.

## Synthesis and crystallization   

All operations were performed under an atmosphere of 99.5% argon further purified by passage through columns of activated BASF catalyst and mol­ecular sieves. Standard Schlenk techniques were employed for all reactions with a double manifold vacuum (0.01 Torr) line. Solutions were transferred *via* stainless steel double-ended needles (cannulas) and glass-covered magnetic stir bars were employed. Cyclo­hepta­triene was distilled from Na/K alloy.

Excess cyclo­hepta­triene was added dropwise to a deep pinkish-red solution of [K(18-crown-6)(THF)_2_][Co(η^4^-C_14_H_10_)_2_] (0.500 g, 0.579 mmol; Brennessel *et al.*, 2002 ▸; Brennessel & Ellis, 2012 ▸) in THF (50 ml, 195 K). The solution was slowly warmed to room temperature, at which point it was deep yellowish brown. After the solvent was removed *in vacuo* and heptane (70 ml) was added, the slurry was filtered. The product was washed with pentane (20 ml) and dried *in vacuo*, yielding a blackish-gray solid [0.292 g, 92%, based on cobalt and using the formulation of (I)]. This product was only characterized by single-crystal X-ray diffraction. Red–brown blocks were grown from a pentane-layered THF solution at 273 K.

## Refinement   

Crystal data, data collection and structure refinement details are summarized in Table 3 ▸. The refinement stalled at *R*_1_ = 0.19, at which point the structure was examined for twinning (Parsons *et al.*, 2003 ▸). Non-merohedral twinning was identified and the data were re-integrated accordingly. Application of twin law [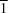 0 0 / 0 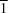 0 / 0.064 0 1], a 180° rotation about reciprocal lattice direction [001], reduced the *R*_1_ residual to its final value of 0.043 (Table 3 ▸). The mass ratio of the twin components refined to 0.5040 (7):0.4960 (7).

**Figure 4:**
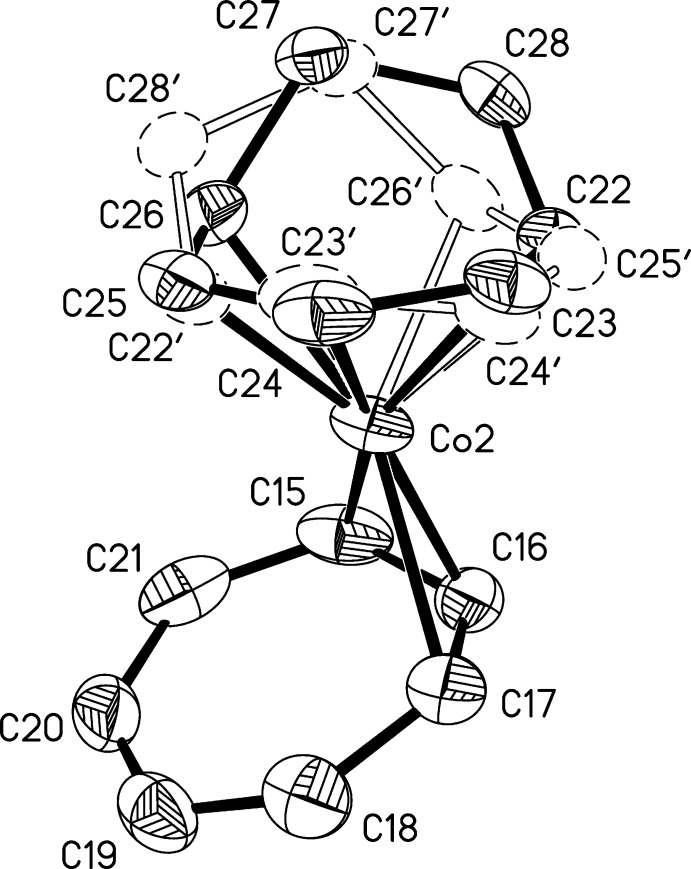
View of the ring ligand disorder. Displacement ellipsoids are shown at the 50% probability level and H atoms have been omitted. The numbering scheme of the minor component of the disorder was chosen to show the mirror-like symmetry that allows both orientations to fit within essentially the same volume.

The η^5^-coordinating ligand C8–C14 is modeled as disordered over two positions with site occupancy ratio 0.699 (5):0.301 (5), such that the ethyl linkage is shifted by one carbon atom (see Fig. 4 ▸). Analogous bond lengths and angles between the two positions of the disordered ring were heavily restrained to be similar. Anisotropic displacement parameters for pairs of proximal atoms from the two components of the disorder were constrained to be equivalent (Sheldrick, 2015 ▸).

H-atom positions of ring-ligand carbon atoms, except those in the minor component of the disorder, were located in a difference map and refined freely. All other H atoms were placed geometrically and treated as riding atoms: methine and *sp* ^2^, C—H = 1.00 Å, and methyl­ene, C—H = 0.99 Å, with *U*_iso_(H) = 1.2*U*_eq_(C).

## Supplementary Material

Crystal structure: contains datablock(s) I, global. DOI: 10.1107/S2056989015003151/bh2504sup1.cif

Structure factors: contains datablock(s) I. DOI: 10.1107/S2056989015003151/bh2504Isup2.hkl

CCDC reference: 1049452

Additional supporting information:  crystallographic information; 3D view; checkCIF report

## Figures and Tables

**Figure 2:**
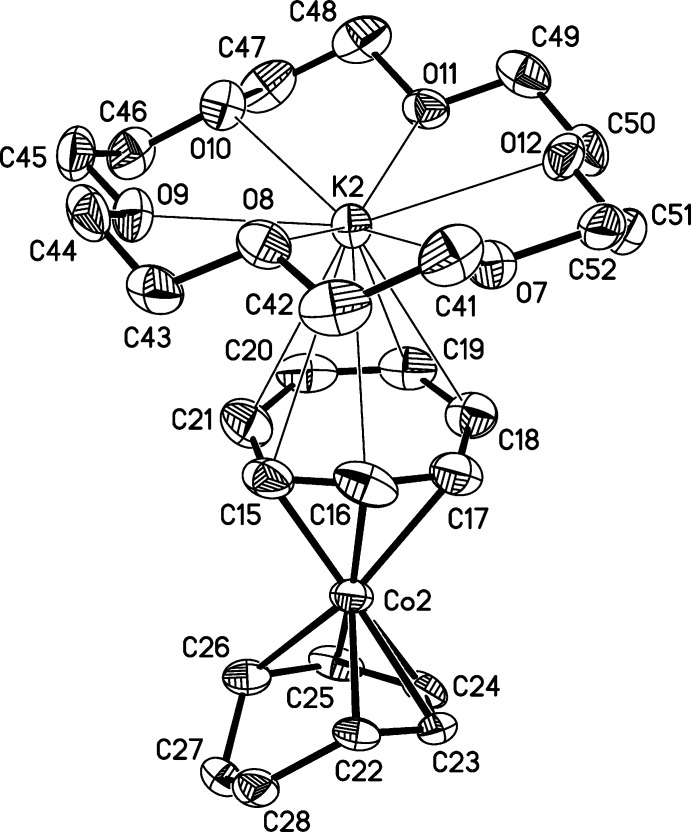
Structure of the second independent mol­ecule of (I), with displacement ellipsoids shown at the 50% probability level. H atoms and the minor component of the disordered ring have been omitted. Thin lines indicate the primarily electrostatic inter­actions between the K^+^ cation and the crown ether and η^3^ ring.

**Figure 3:**
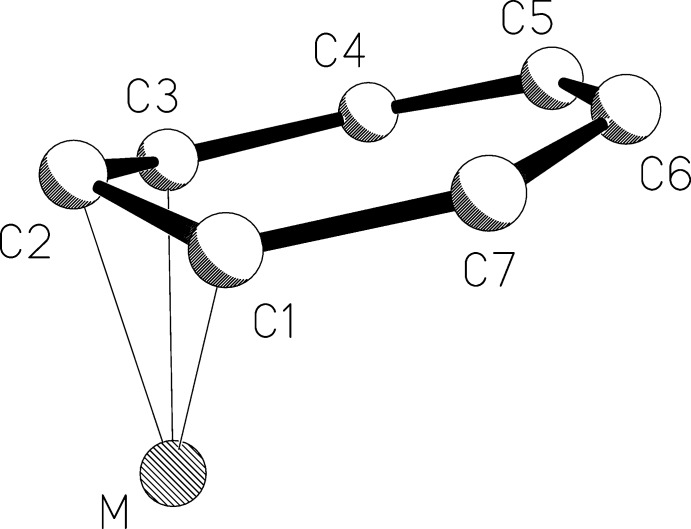
Numbering scheme used for the η^3^-cyclo­hepta­trienyl ligands in Table 2 ▸.

**Table 1:** Selected bond lengths ()

Co1C2	1.924(3)	Co2C16	1.918(3)
Co1C1	2.014(4)	Co2C15	2.005(4)
Co1C9	2.035(3)	Co2C22	2.045(15)
Co1C11	2.040(3)	Co2C24	2.046(13)
Co1C12	2.055(3)	Co2C23	2.092(13)
Co1C10	2.072(3)	Co2C25	2.113(7)
Co1C8	2.105(3)	Co2C26	2.150(5)
Co1C3	2.142(3)	C15C16	1.413(5)
C1C7	1.430(6)	C15C21	1.468(6)
C1C2	1.439(6)	C15K2	3.496(3)
C1K1	3.436(4)	C16C17	1.410(5)
C2C3	1.420(5)	C16K2	3.362(4)
C2K1	3.307(3)	C17C18	1.404(5)
C3C4	1.437(5)	C18C19	1.358(5)
C4C5	1.353(5)	C18K2	3.411(4)
C4K1	3.538(4)	C19C20	1.408(6)
C5C6	1.398(6)	C19K2	3.207(3)
C5K1	3.346(4)	C20C21	1.375(6)
C6C7	1.354(6)	C20K2	3.201(4)
C6K1	3.242(4)	C21K2	3.379(4)
C7K1	3.295(4)	C22C23	1.425(7)
C8C9	1.395(4)	C22C28	1.491(9)
C8C14	1.515(4)	C23C24	1.424(6)
C9C10	1.415(5)	C24C25	1.421(6)
C10C11	1.421(5)	C25C26	1.428(7)
C11C12	1.421(5)	C26C27	1.511(8)
C12C13	1.502(5)	C27C28	1.532(10)
C13C14	1.507(5)		

**Table 2:** Comparison of bond lengths () and fold angles () for selected later transition metal complexes containing ^3^-cycloheptatrienyl ligands, with numbering according to Fig.3 ▸. Fold angles are defined as the angles between the C1C2C3 (allylic) and C1C3C4C5C6C7 (*exo*-diene) mean planes.

Bond	(I)*^*a*^*	(I)*^*b*^*	NEFYII*^*c*^*	SEKJOH*^*d*^*	SEKJIB*^*e*^*
*M*C1	2.014(4)	2.005(4)	2.287(5)	2.252(7)	2.244(5)
*M*C2	1.924(3)	1.918(3)	2.147(6)	2.113(7)	2.124(5)
*M*C3	2.142(3)	2.186(4)	2.213(6)	2.230(7)	2.244(5)
C1C2	1.439(6)	1.413(5)	1.388(8)	1.425(11)	1.439(8)
C2C3	1.420(5)	1.410(5)	1.420(8)	1.432(10)	1.448(8)
C3C4	1.437(5)	1.404(5)	1.446(11)	1.468(10)	1.459(8)
C4C5	1.353(5)	1.358(5)	1.349(12)	1.350(10)	1.348(8)
C5C6	1.398(6)	1.408(6)	1.419(10)	1.429(12)	1.438(9)
C6C7	1.354(6)	1.375(6)	1.338(8)	1.358(11)	1.342(9)
C7C1	1.430(6)	1.468(6)	1.461(8)	1.462(10)	1.455(8)
					
Fold angle	28.0(4)	27.2(4)	29.6	35.8	37.1

Notes: (*a*) (I), ring C1C7; (*b*) (I), ring C15C21; (*c*) [Pd(^3^-C_7_H_7_)(PPh_3_)_2_][BF_4_] (Murahashi *et al.*, 2012 ▸); (*d*) [AsPh_4_][Ru(^3^-C_7_H_7_)(CO)_3_] (Astley *et al.*, 1990 ▸); (*e*) [AsPh_4_][Os(^3^-C_7_H_7_)(CO)_3_] (Astley *et al.*, 1990 ▸).

**Table 3:** Experimental details

Crystal data	
Chemical formula	[K(C_12_H_24_O_6_)][Co(C_7_H_7_)(C_7_H_9_)]
*M*_r_	546.61
Crystal system, space group	Monoclinic, *P*2_1_/*c*
Temperature (K)	173
*a*, *b*, *c* ()	16.3925(19), 17.225(2), 18.678(2)
()	91.6077(19)
*V* (^3^)	5271.8(11)
*Z*	8
Radiation type	Mo *K*
(mm^1^)	0.85
Crystal size (mm)	0.32 0.24 0.16

Data collection	
Diffractometer	Siemens SMART CCD platform
Absorption correction	Multi-scan (*TWINABS*; Sheldrick, 2012 ▸)
*T*_min_, *T*_max_	0.612, 0.746
No. of measured, independent and observed [*I* > 2(*I*)] reflections	85092, 12058, 9117
*R*_int_	0.055
(sin /)_max_ (^1^)	0.649

Refinement	
*R*[*F* ^2^ > 2(*F* ^2^)], *wR*(*F* ^2^), *S*	0.043, 0.084, 1.00
No. of reflections	12058
No. of parameters	728
No. of restraints	45
H-atom treatment	H atoms treated by a mixture of independent and constrained refinement
_max_, _min_ (e ^3^)	0.46, 0.47

Computer programs: *SMART* and *SAINT* (Bruker, 2003 ▸), *SIR97* (Altomare *et al.*, 1999 ▸), *SHELXL2014* (Sheldrick, 2015 ▸) and *SHELXTL* (Sheldrick, 2008 ▸).

## supplementary crystallographic information

### Crystal data

**Table:**

[K(C_12_H_24_O_6_)][Co(C_7_H_7_)(C_7_H_9_)]	*F*(000) = 2320
*M**_r_* = 546.61	*D*_x_ = 1.377 Mg m^−^^3^
Monoclinic, *P*2_1_/*c*	Mo *K*α radiation, λ = 0.71073 Å
*a* = 16.3925 (19) Å	Cell parameters from 3984 reflections
*b* = 17.225 (2) Å	θ = 2.4–27.4°
*c* = 18.678 (2) Å	µ = 0.85 mm^−^^1^
β = 91.6077 (19)°	*T* = 173 K
*V* = 5271.8 (11) Å^3^	Block, red-brown
*Z* = 8	0.32 × 0.24 × 0.16 mm

### Data collection

**Table:**

Siemens SMART CCD platform diffractometer	9117 reflections with *I* > 2σ(*I*)
Radiation source: normal-focus sealed tube	*R*_int_ = 0.055
ω scans	θ_max_ = 27.5°, θ_min_ = 1.2°
Absorption correction: multi-scan (*TWINABS*; Sheldrick, 2012)	*h* = −21→21
*T*_min_ = 0.612, *T*_max_ = 0.746	*k* = 0→22
85092 measured reflections	*l* = 0→24
12058 independent reflections	

### Refinement

**Table:**

Refinement on *F*^2^	Primary atom site location: structure-invariant direct methods
Least-squares matrix: full	Secondary atom site location: difference Fourier map
*R*[*F*^2^ > 2σ(*F*^2^)] = 0.043	Hydrogen site location: mixed
*wR*(*F*^2^) = 0.084	H atoms treated by a mixture of independent and constrained refinement
*S* = 1.00	*w* = 1/[σ^2^(*F*_o_^2^) + (0.0271*P*)^2^ + 2.3296*P*] where *P* = (*F*_o_^2^ + 2*F*_c_^2^)/3
12058 reflections	(Δ/σ)_max_ = 0.001
728 parameters	Δρ_max_ = 0.46 e Å^−^^3^
45 restraints	Δρ_min_ = −0.47 e Å^−^^3^

### Special details

**Table:**

Refinement. The structure was integrated and refined as a non-merohedral twin (Parsons *et al.*, 2003). Application of twin law [-1 0 0 / 0 - 1 0 / 0.064 0 1], a 180° rotation about reciprocal lattice [001], reduced the *R* residual from 19.0% to its final value of 4.3%. The mass ratio of the twin components refined to 0.5039 (7):0.4961 (7).The η^5^-coordinating ligand C8—C14 is modeled as disordered over two positions, 0.697 (5):0.303 (5), such that the ethyl linkage is shifted by one carbon atom. Analogous bond lengths and angles between the two positions of the disordered ring were heavily restrained to be similar. Anisotropic displacement parameters for pairs of proximal atoms from the two components of the disorder were constrained to be equivalent.H atom positions of ring-ligand carbon atoms, except those in the minor component of the disorder, were refined freely. All other H atoms were placed geometrically and treated as riding atoms: methine and *sp*^2^, C—H = 1.00 Å, and methylene, C—H = 0.99 Å, with *U*_iso_(H) = 1.2*U*_eq_(C).

### Fractional atomic coordinates and isotropic or equivalent isotropic displacement parameters (Å^2^)

**Table:**

	*x*	*y*	*z*	*U*_iso_*/*U*_eq_	Occ. (<1)
Co1	0.08386 (3)	0.87509 (2)	0.59699 (2)	0.02870 (10)	
C1	0.1495 (3)	0.8446 (2)	0.5119 (2)	0.0510 (11)	
H1	0.172 (3)	0.881 (2)	0.489 (2)	0.078 (14)*	
C2	0.0624 (3)	0.8404 (2)	0.50036 (18)	0.0433 (9)	
H2	0.035 (2)	0.867 (2)	0.469 (2)	0.059 (12)*	
C3	0.0169 (2)	0.7864 (2)	0.54050 (18)	0.0394 (8)	
H3	−0.039 (2)	0.787 (2)	0.5341 (19)	0.051 (11)*	
C4	0.0432 (2)	0.7125 (2)	0.56839 (19)	0.0443 (9)	
H4	−0.005 (2)	0.688 (2)	0.592 (2)	0.079 (13)*	
C5	0.1172 (3)	0.6777 (2)	0.56901 (19)	0.0500 (10)	
H5	0.124 (2)	0.626 (2)	0.590 (2)	0.076 (13)*	
C6	0.1922 (3)	0.7098 (3)	0.5507 (2)	0.0585 (12)	
H6	0.242 (3)	0.675 (2)	0.561 (2)	0.088 (15)*	
C7	0.2057 (2)	0.7831 (3)	0.5275 (2)	0.0596 (12)	
H7	0.265 (3)	0.800 (2)	0.522 (2)	0.079 (13)*	
C8	0.15714 (18)	0.88005 (18)	0.69126 (16)	0.0293 (7)	
H8	0.1933 (18)	0.8412 (17)	0.7021 (15)	0.031 (8)*	
C9	0.0752 (2)	0.86626 (19)	0.70514 (16)	0.0326 (7)	
H9	0.0561 (17)	0.8191 (17)	0.7215 (15)	0.030 (8)*	
C10	0.0139 (2)	0.9175 (2)	0.67898 (17)	0.0364 (8)	
H10	−0.0423 (19)	0.9061 (16)	0.6853 (15)	0.029 (8)*	
C11	0.0287 (2)	0.97567 (19)	0.62694 (19)	0.0391 (8)	
H11	−0.019 (2)	0.9961 (18)	0.6036 (17)	0.047 (9)*	
C12	0.1052 (2)	0.99269 (17)	0.59661 (18)	0.0357 (7)	
H12	0.101 (2)	1.0225 (19)	0.5523 (18)	0.051 (10)*	
C13	0.1851 (2)	1.00884 (19)	0.63506 (18)	0.0360 (8)	
H13A	0.1932 (18)	1.0645 (18)	0.6471 (15)	0.034 (8)*	
H13B	0.229 (2)	0.9996 (19)	0.604 (2)	0.056 (11)*	
C14	0.1941 (2)	0.95999 (19)	0.70173 (18)	0.0330 (7)	
H14A	0.1658 (17)	0.9837 (16)	0.7411 (15)	0.029 (8)*	
H14B	0.2494 (18)	0.9562 (16)	0.7172 (14)	0.024 (7)*	
Co2	0.39803 (2)	0.38636 (2)	0.59565 (2)	0.03271 (10)	
C15	0.3302 (2)	0.3197 (2)	0.52922 (19)	0.0440 (9)	
H15	0.276 (2)	0.333 (2)	0.518 (2)	0.067 (12)*	
C16	0.3926 (3)	0.3615 (2)	0.49555 (17)	0.0451 (9)	
H16	0.381 (2)	0.402 (2)	0.4625 (18)	0.054 (10)*	
C17	0.4764 (2)	0.3482 (2)	0.50970 (19)	0.0438 (9)	
H17	0.519 (2)	0.3912 (19)	0.4897 (18)	0.056 (10)*	
C18	0.5157 (2)	0.2777 (2)	0.52637 (19)	0.0439 (9)	
H18	0.579 (2)	0.280 (2)	0.525 (2)	0.069 (12)*	
C19	0.4846 (2)	0.2077 (2)	0.54489 (18)	0.0455 (9)	
H19	0.527 (2)	0.162 (2)	0.5528 (17)	0.057 (11)*	
C20	0.4028 (3)	0.1907 (2)	0.55977 (19)	0.0480 (9)	
H20	0.392 (2)	0.1417 (19)	0.5780 (17)	0.044 (9)*	
C21	0.3370 (3)	0.2401 (3)	0.5569 (2)	0.0549 (11)	
H21	0.291 (2)	0.224 (2)	0.572 (2)	0.063 (13)*	
C22	0.3724 (5)	0.5020 (8)	0.6053 (8)	0.0308 (11)	0.699 (5)
H22	0.3723	0.5303	0.5585	0.037*	0.699 (5)
C23	0.4523 (5)	0.4885 (7)	0.6346 (8)	0.031 (2)	0.699 (5)
H23	0.4942	0.5245	0.6242	0.038*	0.699 (5)
C24	0.4724 (5)	0.4234 (9)	0.6786 (8)	0.0380 (13)	0.699 (5)
H24	0.5286	0.4150	0.6900	0.046*	0.699 (5)
C25	0.4159 (4)	0.3698 (5)	0.7071 (4)	0.0332 (15)	0.699 (5)
H25	0.4345	0.3271	0.7352	0.040*	0.699 (5)
C26	0.3307 (3)	0.3810 (3)	0.6927 (3)	0.0344 (11)	0.699 (5)
H26	0.2949	0.3346	0.6986	0.041*	0.699 (5)
C27	0.2936 (6)	0.4589 (6)	0.7100 (3)	0.0303 (12)	0.699 (5)
H27A	0.3241	0.4825	0.7510	0.036*	0.699 (5)
H27B	0.2363	0.4515	0.7240	0.036*	0.699 (5)
C28	0.2960 (3)	0.5137 (3)	0.6455 (3)	0.0335 (10)	0.699 (5)
H28A	0.2481	0.5038	0.6133	0.040*	0.699 (5)
H28B	0.2931	0.5681	0.6621	0.040*	0.699 (5)
C22'	0.4013 (10)	0.3665 (13)	0.6930 (10)	0.0332 (15)	0.301 (5)
H22'	0.4212	0.3132	0.7056	0.040*	0.301 (5)
C23'	0.4652 (10)	0.422 (2)	0.6851 (19)	0.0380 (13)	0.301 (5)
H23'	0.5118	0.4215	0.7165	0.046*	0.301 (5)
C24'	0.4570 (11)	0.4773 (19)	0.629 (2)	0.031 (2)	0.301 (5)
H24'	0.5062	0.4977	0.6105	0.038*	0.301 (5)
C25'	0.3825 (11)	0.506 (2)	0.5966 (19)	0.0308 (11)	0.301 (5)
H25'	0.3834	0.5452	0.5613	0.037*	0.301 (5)
C26'	0.3069 (6)	0.4736 (6)	0.6182 (5)	0.0335 (10)	0.301 (5)
H26C	0.2592	0.4781	0.5839	0.040*	0.301 (5)
C27'	0.2893 (16)	0.4648 (14)	0.6962 (8)	0.0303 (12)	0.301 (5)
H27C	0.2297	0.4678	0.7028	0.036*	0.301 (5)
H27D	0.3154	0.5078	0.7236	0.036*	0.301 (5)
C28'	0.3216 (8)	0.3867 (7)	0.7254 (6)	0.0344 (11)	0.301 (5)
H28C	0.3287	0.3899	0.7781	0.041*	0.301 (5)
H28D	0.2812	0.3454	0.7142	0.041*	0.301 (5)
K1	0.11102 (4)	0.69805 (3)	0.39075 (3)	0.02821 (14)	
O1	0.21703 (13)	0.79524 (13)	0.31156 (12)	0.0361 (5)	
C29	0.1870 (2)	0.87284 (18)	0.3082 (2)	0.0439 (8)	
H29A	0.2247	0.9058	0.2809	0.053*	
H29B	0.1834	0.8944	0.3571	0.053*	
C30	0.1046 (2)	0.87240 (18)	0.27208 (18)	0.0433 (8)	
H30A	0.0854	0.9263	0.2641	0.052*	
H30B	0.1071	0.8461	0.2251	0.052*	
O2	0.05022 (13)	0.83223 (12)	0.31708 (10)	0.0332 (5)	
C31	−0.0317 (2)	0.83326 (19)	0.28937 (18)	0.0409 (8)	
H31A	−0.0343	0.8096	0.2410	0.049*	
H31B	−0.0515	0.8874	0.2854	0.049*	
C32	−0.08384 (19)	0.78852 (18)	0.33875 (18)	0.0405 (8)	
H32A	−0.0788	0.8103	0.3877	0.049*	
H32B	−0.1417	0.7919	0.3225	0.049*	
O3	−0.05789 (12)	0.71010 (13)	0.33890 (12)	0.0378 (5)	
C33	−0.10669 (19)	0.6611 (2)	0.38195 (18)	0.0422 (8)	
H33A	−0.1649	0.6653	0.3667	0.051*	
H33B	−0.1012	0.6766	0.4329	0.051*	
C34	−0.0775 (2)	0.5799 (2)	0.37251 (19)	0.0447 (9)	
H34A	−0.1157	0.5431	0.3948	0.054*	
H34B	−0.0749	0.5674	0.3209	0.054*	
O4	0.00127 (13)	0.57276 (11)	0.40546 (13)	0.0388 (5)	
C35	0.0343 (2)	0.49680 (19)	0.3986 (2)	0.0513 (10)	
H35A	0.0403	0.4840	0.3473	0.062*	
H35B	−0.0028	0.4583	0.4197	0.062*	
C36	0.1150 (2)	0.49405 (19)	0.4361 (2)	0.0499 (10)	
H36A	0.1102	0.5144	0.4854	0.060*	
H36B	0.1342	0.4396	0.4393	0.060*	
O5	0.17146 (13)	0.53887 (12)	0.39880 (12)	0.0364 (5)	
C37	0.2495 (2)	0.5416 (2)	0.43407 (18)	0.0426 (9)	
H37A	0.2700	0.4883	0.4426	0.051*	
H37B	0.2453	0.5679	0.4809	0.051*	
C38	0.3067 (2)	0.5849 (2)	0.38815 (18)	0.0414 (8)	
H38A	0.3626	0.5832	0.4095	0.050*	
H38B	0.3077	0.5612	0.3399	0.050*	
O6	0.27920 (13)	0.66290 (12)	0.38309 (12)	0.0362 (5)	
C39	0.32695 (19)	0.7087 (2)	0.33672 (18)	0.0407 (8)	
H39A	0.3208	0.6896	0.2869	0.049*	
H39B	0.3853	0.7056	0.3515	0.049*	
C40	0.29782 (19)	0.7908 (2)	0.34127 (18)	0.0407 (8)	
H40A	0.2984	0.8079	0.3919	0.049*	
H40B	0.3344	0.8254	0.3145	0.049*	
K2	0.39810 (4)	0.20452 (3)	0.38877 (3)	0.02898 (14)	
O7	0.45283 (14)	0.33192 (12)	0.30801 (11)	0.0368 (5)	
C41	0.3973 (2)	0.3681 (2)	0.25962 (19)	0.0485 (9)	
H41A	0.3914	0.3367	0.2153	0.058*	
H41B	0.4177	0.4201	0.2466	0.058*	
C42	0.3170 (2)	0.37518 (19)	0.2941 (2)	0.0476 (9)	
H42A	0.3236	0.4036	0.3400	0.057*	
H42B	0.2786	0.4047	0.2625	0.057*	
O8	0.28521 (13)	0.29987 (12)	0.30686 (12)	0.0380 (5)	
C43	0.20880 (19)	0.3019 (2)	0.34089 (19)	0.0425 (9)	
H43A	0.1704	0.3360	0.3137	0.051*	
H43B	0.2159	0.3229	0.3900	0.051*	
C44	0.17541 (19)	0.2215 (2)	0.34377 (19)	0.0450 (9)	
H44A	0.1190	0.2226	0.3613	0.054*	
H44B	0.1743	0.1981	0.2954	0.054*	
O9	0.22655 (13)	0.17710 (13)	0.39114 (12)	0.0390 (5)	
C45	0.1980 (2)	0.0998 (2)	0.4002 (2)	0.0471 (9)	
H45A	0.1895	0.0747	0.3529	0.056*	
H45B	0.1452	0.1003	0.4248	0.056*	
C46	0.2594 (2)	0.0557 (2)	0.44361 (19)	0.0460 (9)	
H46A	0.2707	0.0826	0.4897	0.055*	
H46B	0.2385	0.0031	0.4540	0.055*	
O10	0.33194 (13)	0.05044 (12)	0.40421 (12)	0.0372 (5)	
C47	0.3896 (2)	−0.00261 (18)	0.4340 (2)	0.0499 (10)	
H47A	0.3662	−0.0556	0.4345	0.060*	
H47B	0.4042	0.0124	0.4839	0.060*	
C48	0.4635 (2)	−0.00123 (19)	0.3895 (2)	0.0508 (10)	
H48A	0.5010	−0.0437	0.4042	0.061*	
H48B	0.4477	−0.0086	0.3384	0.061*	
O11	0.50257 (13)	0.07131 (11)	0.39930 (12)	0.0369 (5)	
C49	0.5787 (2)	0.0755 (2)	0.36519 (19)	0.0452 (9)	
H49A	0.5716	0.0619	0.3139	0.054*	
H49B	0.6176	0.0381	0.3876	0.054*	
C50	0.61118 (19)	0.1558 (2)	0.37255 (19)	0.0449 (9)	
H50A	0.6114	0.1720	0.4234	0.054*	
H50B	0.6679	0.1580	0.3557	0.054*	
O12	0.56028 (12)	0.20619 (13)	0.33055 (12)	0.0370 (5)	
C51	0.5866 (2)	0.2849 (2)	0.33212 (19)	0.0451 (9)	
H51A	0.6443	0.2884	0.3181	0.054*	
H51B	0.5822	0.3063	0.3811	0.054*	
C52	0.5337 (2)	0.3295 (2)	0.28107 (18)	0.0430 (9)	
H52A	0.5551	0.3828	0.2759	0.052*	
H52B	0.5330	0.3042	0.2334	0.052*	

### Atomic displacement parameters (Å^2^)

**Table:**

	*U*^11^	*U*^22^	*U*^33^	*U*^12^	*U*^13^	*U*^23^
Co1	0.0393 (2)	0.02537 (19)	0.02133 (19)	−0.00096 (19)	−0.00152 (18)	−0.00292 (16)
C1	0.077 (3)	0.050 (2)	0.0272 (19)	−0.034 (2)	0.0239 (19)	−0.0144 (17)
C2	0.074 (3)	0.0300 (18)	0.0251 (17)	0.0044 (19)	−0.0124 (18)	−0.0030 (14)
C3	0.0346 (18)	0.049 (2)	0.0348 (18)	0.0014 (17)	−0.0005 (15)	−0.0142 (15)
C4	0.060 (2)	0.0315 (18)	0.042 (2)	−0.0090 (18)	0.0182 (18)	−0.0024 (15)
C5	0.081 (3)	0.0335 (19)	0.0358 (19)	0.016 (2)	0.002 (2)	−0.0059 (15)
C6	0.054 (2)	0.080 (3)	0.041 (2)	0.019 (3)	−0.0118 (19)	−0.026 (2)
C7	0.037 (2)	0.097 (4)	0.045 (2)	−0.016 (2)	0.0044 (18)	−0.038 (2)
C8	0.0373 (16)	0.0262 (17)	0.0244 (16)	0.0058 (15)	−0.0027 (13)	0.0016 (12)
C9	0.0414 (18)	0.0349 (17)	0.0219 (15)	−0.0082 (15)	0.0068 (14)	−0.0005 (13)
C10	0.0299 (17)	0.0422 (19)	0.0370 (19)	−0.0042 (15)	−0.0006 (15)	−0.0148 (16)
C11	0.0356 (18)	0.0309 (18)	0.050 (2)	0.0050 (15)	−0.0118 (16)	−0.0138 (15)
C12	0.050 (2)	0.0246 (14)	0.0324 (17)	−0.0010 (15)	−0.0049 (16)	−0.0002 (14)
C13	0.0433 (19)	0.0273 (18)	0.0374 (19)	−0.0060 (15)	0.0036 (16)	0.0005 (14)
C14	0.0305 (17)	0.0375 (18)	0.0309 (18)	−0.0008 (15)	−0.0013 (14)	−0.0076 (14)
Co2	0.0306 (2)	0.0312 (2)	0.0362 (2)	0.00488 (19)	−0.00151 (19)	−0.01252 (18)
C15	0.0350 (18)	0.056 (2)	0.041 (2)	0.0139 (17)	−0.0100 (16)	−0.0203 (18)
C16	0.069 (3)	0.0418 (19)	0.0245 (16)	0.020 (2)	0.0061 (17)	0.0050 (14)
C17	0.055 (2)	0.0360 (19)	0.041 (2)	0.0036 (18)	0.0107 (18)	−0.0036 (16)
C18	0.044 (2)	0.048 (2)	0.0394 (19)	0.0062 (18)	−0.0088 (17)	−0.0044 (16)
C19	0.057 (2)	0.041 (2)	0.0379 (19)	0.0158 (19)	−0.0108 (17)	−0.0020 (15)
C20	0.079 (3)	0.0316 (18)	0.0331 (18)	0.004 (2)	0.0024 (19)	−0.0010 (14)
C21	0.057 (3)	0.071 (3)	0.038 (2)	−0.002 (2)	0.0151 (19)	−0.009 (2)
C22	0.039 (3)	0.027 (2)	0.027 (4)	0.000 (2)	0.001 (2)	−0.0030 (18)
C23	0.0296 (18)	0.027 (4)	0.037 (3)	−0.004 (2)	−0.0002 (18)	−0.014 (3)
C24	0.029 (2)	0.051 (2)	0.034 (3)	0.013 (2)	−0.003 (2)	−0.013 (2)
C25	0.048 (3)	0.0353 (19)	0.015 (3)	0.018 (3)	−0.014 (2)	−0.008 (2)
C26	0.044 (2)	0.035 (2)	0.024 (3)	−0.0001 (19)	0.002 (3)	−0.002 (3)
C27	0.0300 (19)	0.036 (2)	0.025 (3)	0.0070 (16)	0.002 (3)	−0.004 (3)
C28	0.033 (2)	0.024 (2)	0.043 (3)	0.008 (2)	−0.003 (2)	−0.0027 (18)
C22'	0.048 (3)	0.0353 (19)	0.015 (3)	0.018 (3)	−0.014 (2)	−0.008 (2)
C23'	0.029 (2)	0.051 (2)	0.034 (3)	0.013 (2)	−0.003 (2)	−0.013 (2)
C24'	0.0296 (18)	0.027 (4)	0.037 (3)	−0.004 (2)	−0.0002 (18)	−0.014 (3)
C25'	0.039 (3)	0.027 (2)	0.027 (4)	0.000 (2)	0.001 (2)	−0.0030 (18)
C26'	0.033 (2)	0.024 (2)	0.043 (3)	0.008 (2)	−0.003 (2)	−0.0027 (18)
C27'	0.0300 (19)	0.036 (2)	0.025 (3)	0.0070 (16)	0.002 (3)	−0.004 (3)
C28'	0.044 (2)	0.035 (2)	0.024 (3)	−0.0001 (19)	0.002 (3)	−0.002 (3)
K1	0.0271 (3)	0.0252 (3)	0.0322 (4)	−0.0006 (3)	−0.0016 (3)	0.0035 (3)
O1	0.0371 (12)	0.0336 (12)	0.0376 (13)	−0.0071 (10)	0.0006 (10)	0.0006 (10)
C29	0.060 (2)	0.0255 (17)	0.047 (2)	−0.0121 (17)	0.0115 (17)	0.0086 (15)
C30	0.063 (2)	0.0337 (18)	0.0334 (17)	0.0069 (18)	0.0004 (17)	0.0099 (14)
O2	0.0384 (12)	0.0314 (12)	0.0294 (12)	0.0044 (10)	−0.0043 (9)	0.0045 (9)
C31	0.048 (2)	0.0359 (18)	0.0375 (19)	0.0170 (16)	−0.0203 (16)	−0.0051 (15)
C32	0.0334 (17)	0.046 (2)	0.0422 (19)	0.0128 (16)	−0.0044 (15)	−0.0109 (16)
O3	0.0303 (11)	0.0411 (13)	0.0421 (13)	0.0051 (10)	0.0023 (10)	−0.0026 (11)
C33	0.0238 (16)	0.063 (2)	0.0398 (19)	−0.0076 (16)	−0.0015 (14)	0.0025 (16)
C34	0.0392 (19)	0.054 (2)	0.0405 (19)	−0.0227 (18)	0.0011 (16)	0.0024 (16)
O4	0.0400 (12)	0.0293 (11)	0.0470 (13)	−0.0078 (10)	0.0013 (11)	−0.0035 (11)
C35	0.058 (2)	0.0286 (17)	0.069 (3)	−0.0108 (17)	0.026 (2)	−0.0085 (19)
C36	0.065 (2)	0.0269 (17)	0.059 (2)	0.0092 (18)	0.024 (2)	0.0113 (16)
O5	0.0420 (13)	0.0352 (12)	0.0321 (12)	0.0052 (10)	0.0036 (10)	0.0062 (10)
C37	0.055 (2)	0.0396 (19)	0.0325 (18)	0.0239 (17)	−0.0056 (16)	0.0003 (15)
C38	0.0344 (18)	0.050 (2)	0.0397 (19)	0.0196 (16)	−0.0059 (15)	−0.0092 (16)
O6	0.0281 (11)	0.0430 (13)	0.0377 (13)	0.0047 (10)	0.0047 (10)	−0.0008 (10)
C39	0.0262 (16)	0.062 (2)	0.0341 (18)	−0.0014 (16)	0.0058 (13)	−0.0067 (17)
C40	0.0323 (17)	0.053 (2)	0.0370 (19)	−0.0142 (17)	0.0071 (14)	−0.0057 (17)
K2	0.0277 (3)	0.0263 (3)	0.0329 (4)	0.0028 (3)	0.0007 (3)	0.0054 (3)
O7	0.0481 (13)	0.0332 (12)	0.0294 (12)	−0.0045 (10)	0.0067 (10)	0.0041 (10)
C41	0.068 (2)	0.0327 (17)	0.044 (2)	−0.0093 (19)	−0.0025 (19)	0.0153 (15)
C42	0.067 (2)	0.0256 (18)	0.049 (2)	0.0151 (18)	−0.0116 (19)	0.0085 (15)
O8	0.0407 (12)	0.0324 (12)	0.0408 (14)	0.0103 (11)	0.0019 (10)	0.0003 (10)
C43	0.0360 (17)	0.048 (2)	0.043 (2)	0.0218 (17)	−0.0104 (15)	−0.0078 (17)
C44	0.0229 (16)	0.071 (3)	0.041 (2)	0.0118 (16)	−0.0020 (14)	−0.0047 (18)
O9	0.0272 (11)	0.0472 (13)	0.0423 (14)	−0.0015 (10)	−0.0025 (10)	0.0040 (11)
C45	0.0318 (17)	0.065 (2)	0.045 (2)	−0.0154 (17)	0.0060 (16)	−0.0024 (19)
C46	0.054 (2)	0.042 (2)	0.042 (2)	−0.0183 (18)	0.0070 (17)	0.0099 (16)
O10	0.0398 (12)	0.0356 (12)	0.0360 (12)	−0.0014 (10)	−0.0010 (10)	0.0122 (11)
C47	0.065 (2)	0.0254 (17)	0.058 (2)	−0.0029 (18)	−0.020 (2)	0.0122 (15)
C48	0.056 (2)	0.0241 (17)	0.071 (3)	0.0156 (17)	−0.016 (2)	−0.0060 (17)
O11	0.0375 (12)	0.0332 (12)	0.0399 (12)	0.0094 (10)	−0.0002 (10)	−0.0050 (11)
C49	0.043 (2)	0.053 (2)	0.0396 (19)	0.0237 (18)	−0.0013 (17)	−0.0009 (16)
C50	0.0219 (16)	0.074 (3)	0.0387 (19)	0.0068 (17)	−0.0033 (14)	−0.0057 (17)
O12	0.0270 (11)	0.0403 (12)	0.0434 (13)	−0.0053 (10)	−0.0060 (9)	−0.0035 (10)
C51	0.0370 (18)	0.054 (2)	0.044 (2)	−0.0175 (17)	0.0068 (16)	−0.0136 (17)
C52	0.053 (2)	0.0366 (19)	0.040 (2)	−0.0177 (17)	0.0147 (17)	−0.0064 (16)

### Geometric parameters (Å, º)

**Table:**

Co1—C2	1.924 (3)	C26'—H26C	1.0000
Co1—C1	2.014 (4)	C27'—C28'	1.538 (15)
Co1—C9	2.035 (3)	C27'—H27C	0.9900
Co1—C11	2.040 (3)	C27'—H27D	0.9900
Co1—C12	2.055 (3)	C28'—H28C	0.9900
Co1—C10	2.072 (3)	C28'—H28D	0.9900
Co1—C8	2.105 (3)	K1—O4	2.828 (2)
Co1—C3	2.142 (3)	K1—O6	2.830 (2)
C1—C7	1.430 (6)	K1—O2	2.855 (2)
C1—C2	1.439 (6)	K1—O1	2.856 (2)
C1—K1	3.436 (4)	K1—O3	2.915 (2)
C1—H1	0.85 (4)	K1—O5	2.918 (2)
C2—C3	1.420 (5)	O1—C40	1.424 (4)
C2—K1	3.307 (3)	O1—C29	1.425 (4)
C2—H2	0.85 (4)	C29—C30	1.493 (5)
C3—C4	1.437 (5)	C29—H29A	0.9900
C3—H3	0.91 (4)	C29—H29B	0.9900
C4—C5	1.353 (5)	C30—O2	1.422 (4)
C4—K1	3.538 (4)	C30—H30A	0.9900
C4—H4	1.01 (4)	C30—H30B	0.9900
C5—C6	1.398 (6)	O2—C31	1.426 (4)
C5—K1	3.346 (4)	C31—C32	1.489 (5)
C5—H5	0.98 (4)	C31—H31A	0.9900
C6—C7	1.354 (6)	C31—H31B	0.9900
C6—K1	3.242 (4)	C32—O3	1.416 (3)
C6—H6	1.04 (4)	C32—H32A	0.9900
C7—K1	3.295 (4)	C32—H32B	0.9900
C7—H7	1.03 (4)	O3—C33	1.427 (4)
C8—C9	1.395 (4)	C33—C34	1.491 (5)
C8—C14	1.515 (4)	C33—H33A	0.9900
C8—H8	0.91 (3)	C33—H33B	0.9900
C9—C10	1.415 (5)	C34—O4	1.419 (4)
C9—H9	0.93 (3)	C34—H34A	0.9900
C10—C11	1.421 (5)	C34—H34B	0.9900
C10—H10	0.95 (3)	O4—C35	1.423 (4)
C11—C12	1.421 (5)	C35—C36	1.480 (5)
C11—H11	0.95 (3)	C35—H35A	0.9900
C12—C13	1.502 (5)	C35—H35B	0.9900
C12—H12	0.97 (3)	C36—O5	1.406 (4)
C13—C14	1.507 (5)	C36—H36A	0.9900
C13—H13A	0.99 (3)	C36—H36B	0.9900
C13—H13B	0.95 (4)	O5—C37	1.423 (4)
C14—H14A	0.97 (3)	C37—C38	1.489 (5)
C14—H14B	0.95 (3)	C37—H37A	0.9900
Co2—C22'	1.850 (18)	C37—H37B	0.9900
Co2—C16	1.918 (3)	C38—O6	1.419 (4)
Co2—C24'	1.93 (3)	C38—H38A	0.9900
Co2—C15	2.005 (4)	C38—H38B	0.9900
Co2—C22	2.045 (15)	O6—C39	1.423 (4)
Co2—C24	2.046 (13)	C39—C40	1.496 (4)
Co2—C23'	2.07 (3)	C39—H39A	0.9900
Co2—C25'	2.07 (3)	C39—H39B	0.9900
Co2—C23	2.092 (13)	C40—H40A	0.9900
Co2—C25	2.113 (7)	C40—H40B	0.9900
Co2—C26	2.150 (5)	K2—O7	2.823 (2)
Co2—C26'	2.167 (10)	K2—O9	2.853 (2)
C15—C16	1.413 (5)	K2—O11	2.867 (2)
C15—C21	1.468 (6)	K2—O8	2.882 (2)
C15—K2	3.496 (3)	K2—O10	2.885 (2)
C15—H15	0.94 (4)	K2—O12	2.901 (2)
C16—C17	1.410 (5)	O7—C41	1.410 (4)
C16—K2	3.362 (4)	O7—C52	1.432 (4)
C16—H16	0.95 (3)	C41—C42	1.487 (5)
C17—C18	1.404 (5)	C41—H41A	0.9900
C17—H17	1.09 (4)	C41—H41B	0.9900
C18—C19	1.358 (5)	C42—O8	1.420 (4)
C18—K2	3.411 (4)	C42—H42A	0.9900
C18—H18	1.03 (4)	C42—H42B	0.9900
C19—C20	1.408 (6)	O8—C43	1.421 (4)
C19—K2	3.207 (3)	C43—C44	1.490 (5)
C19—H19	1.05 (4)	C43—H43A	0.9900
C20—C21	1.375 (6)	C43—H43B	0.9900
C20—K2	3.201 (4)	C44—O9	1.425 (4)
C20—H20	0.93 (3)	C44—H44A	0.9900
C21—K2	3.379 (4)	C44—H44B	0.9900
C21—H21	0.85 (4)	O9—C45	1.424 (4)
C22—C23	1.425 (7)	C45—C46	1.484 (5)
C22—C28	1.491 (9)	C45—H45A	0.9900
C22—H22	1.0000	C45—H45B	0.9900
C23—C24	1.424 (6)	C46—O10	1.419 (4)
C23—H23	0.9500	C46—H46A	0.9900
C24—C25	1.421 (6)	C46—H46B	0.9900
C24—H24	0.9500	O10—C47	1.418 (4)
C25—C26	1.428 (7)	C47—C48	1.488 (5)
C25—H25	0.9500	C47—H47A	0.9900
C26—C27	1.511 (8)	C47—H47B	0.9900
C26—H26	1.0000	C48—O11	1.414 (4)
C27—C28	1.532 (10)	C48—H48A	0.9900
C27—H27A	0.9900	C48—H48B	0.9900
C27—H27B	0.9900	O11—C49	1.419 (4)
C28—H28A	0.9900	C49—C50	1.487 (5)
C28—H28B	0.9900	C49—H49A	0.9900
C22'—C23'	1.424 (10)	C49—H49B	0.9900
C22'—C28'	1.497 (11)	C50—O12	1.424 (4)
C22'—H22'	1.0000	C50—H50A	0.9900
C23'—C24'	1.428 (9)	C50—H50B	0.9900
C23'—H23'	0.9500	O12—C51	1.422 (4)
C24'—C25'	1.430 (9)	C51—C52	1.485 (5)
C24'—H24'	0.9500	C51—H51A	0.9900
C25'—C26'	1.424 (10)	C51—H51B	0.9900
C25'—H25'	0.9500	C52—H52A	0.9900
C26'—C27'	1.502 (10)	C52—H52B	0.9900
			
C2—Co1—C1	42.78 (16)	O1—K1—O3	111.86 (7)
C2—Co1—C9	153.16 (15)	O4—K1—O5	59.59 (6)
C1—Co1—C9	144.86 (17)	O6—K1—O5	58.11 (6)
C2—Co1—C11	116.74 (15)	O2—K1—O5	154.03 (7)
C1—Co1—C11	133.54 (17)	O1—K1—O5	111.53 (6)
C9—Co1—C11	75.37 (14)	O3—K1—O5	113.71 (6)
C2—Co1—C12	109.21 (14)	O4—K1—C6	101.77 (11)
C1—Co1—C12	99.20 (15)	O6—K1—C6	71.64 (9)
C9—Co1—C12	95.37 (14)	O2—K1—C6	121.46 (10)
C11—Co1—C12	40.60 (13)	O1—K1—C6	101.57 (11)
C2—Co1—C10	135.42 (16)	O3—K1—C6	131.52 (9)
C1—Co1—C10	173.91 (17)	O5—K1—C6	83.19 (10)
C9—Co1—C10	40.30 (13)	O4—K1—C7	123.43 (10)
C11—Co1—C10	40.43 (14)	O6—K1—C7	72.30 (9)
C12—Co1—C10	75.68 (14)	O2—K1—C7	99.46 (11)
C2—Co1—C8	151.90 (15)	O1—K1—C7	81.99 (10)
C1—Co1—C8	111.47 (16)	O3—K1—C7	130.41 (9)
C9—Co1—C8	39.34 (12)	O5—K1—C7	103.05 (11)
C11—Co1—C8	89.04 (13)	C6—K1—C7	23.89 (11)
C12—Co1—C8	82.53 (13)	O4—K1—C2	109.94 (9)
C10—Co1—C8	71.48 (13)	O6—K1—C2	116.23 (9)
C2—Co1—C3	40.45 (15)	O2—K1—C2	67.10 (7)
C1—Co1—C3	72.70 (14)	O1—K1—C2	92.66 (8)
C9—Co1—C3	112.76 (14)	O3—K1—C2	84.82 (8)
C11—Co1—C3	121.05 (13)	O5—K1—C2	138.81 (8)
C12—Co1—C3	141.81 (14)	C6—K1—C2	58.85 (10)
C10—Co1—C3	109.20 (13)	C7—K1—C2	46.11 (11)
C8—Co1—C3	135.52 (13)	O4—K1—C5	79.94 (9)
C7—C1—C2	128.7 (4)	O6—K1—C5	91.46 (9)
C7—C1—Co1	112.8 (3)	O2—K1—C5	124.37 (8)
C2—C1—Co1	65.3 (2)	O1—K1—C5	125.07 (9)
C7—C1—K1	72.2 (2)	O3—K1—C5	109.88 (9)
C2—C1—K1	72.7 (2)	O5—K1—C5	81.36 (8)
Co1—C1—K1	128.27 (15)	C6—K1—C5	24.44 (10)
C7—C1—H1	111 (3)	C7—K1—C5	43.50 (11)
C2—C1—H1	114 (3)	C2—K1—C5	57.50 (9)
Co1—C1—H1	117 (3)	O4—K1—C1	127.02 (8)
K1—C1—H1	107 (3)	O6—K1—C1	91.69 (9)
C3—C2—C1	119.1 (4)	O2—K1—C1	77.15 (9)
C3—C2—Co1	78.0 (2)	O1—K1—C1	78.89 (8)
C1—C2—Co1	71.9 (2)	O3—K1—C1	108.74 (9)
C3—C2—K1	88.8 (2)	O5—K1—C1	126.92 (10)
C1—C2—K1	82.8 (2)	C6—K1—C1	44.31 (12)
Co1—C2—K1	140.36 (17)	C7—K1—C1	24.41 (11)
C3—C2—H2	116 (3)	C2—K1—C1	24.54 (10)
C1—C2—H2	125 (3)	C5—K1—C1	54.68 (9)
Co1—C2—H2	123 (3)	O4—K1—C4	75.23 (8)
K1—C2—H2	96 (2)	O6—K1—C4	113.25 (8)
C2—C3—C4	128.0 (3)	O2—K1—C4	106.28 (8)
C2—C3—Co1	61.50 (19)	O1—K1—C4	131.02 (7)
C4—C3—Co1	108.0 (2)	O3—K1—C4	89.20 (8)
C2—C3—H3	117 (2)	O5—K1—C4	97.64 (7)
C4—C3—H3	111 (2)	C6—K1—C4	42.52 (10)
Co1—C3—H3	123 (2)	C7—K1—C4	52.78 (10)
C5—C4—C3	130.9 (4)	C2—K1—C4	43.91 (8)
C5—C4—K1	70.8 (2)	C5—K1—C4	22.45 (9)
C3—C4—K1	79.7 (2)	C1—K1—C4	52.22 (8)
C5—C4—H4	121 (2)	C40—O1—C29	112.5 (3)
C3—C4—H4	108 (2)	C40—O1—K1	109.87 (18)
K1—C4—H4	131 (2)	C29—O1—K1	111.03 (17)
C4—C5—C6	128.1 (4)	O1—C29—C30	108.8 (3)
C4—C5—K1	86.7 (2)	O1—C29—H29A	109.9
C6—C5—K1	73.6 (2)	C30—C29—H29A	109.9
C4—C5—H5	119 (2)	O1—C29—H29B	109.9
C6—C5—H5	112 (2)	C30—C29—H29B	109.9
K1—C5—H5	120 (2)	H29A—C29—H29B	108.3
C7—C6—C5	126.8 (4)	O2—C30—C29	108.1 (2)
C7—C6—K1	80.2 (2)	O2—C30—H30A	110.1
C5—C6—K1	82.0 (2)	C29—C30—H30A	110.1
C7—C6—H6	118 (2)	O2—C30—H30B	110.1
C5—C6—H6	115 (2)	C29—C30—H30B	110.1
K1—C6—H6	116 (2)	H30A—C30—H30B	108.4
C6—C7—C1	130.2 (4)	C30—O2—C31	112.3 (2)
C6—C7—K1	75.9 (2)	C30—O2—K1	117.54 (18)
C1—C7—K1	83.3 (2)	C31—O2—K1	119.83 (19)
C6—C7—H7	117 (2)	O2—C31—C32	108.6 (3)
C1—C7—H7	112 (2)	O2—C31—H31A	110.0
K1—C7—H7	119 (2)	C32—C31—H31A	110.0
C9—C8—C14	121.0 (3)	O2—C31—H31B	110.0
C9—C8—Co1	67.60 (18)	C32—C31—H31B	110.0
C14—C8—Co1	111.2 (2)	H31A—C31—H31B	108.3
C9—C8—H8	117.1 (18)	O3—C32—C31	108.5 (3)
C14—C8—H8	112.5 (19)	O3—C32—H32A	110.0
Co1—C8—H8	120.5 (18)	C31—C32—H32A	110.0
C8—C9—C10	120.6 (3)	O3—C32—H32B	110.0
C8—C9—Co1	73.07 (18)	C31—C32—H32B	110.0
C10—C9—Co1	71.29 (18)	H32A—C32—H32B	108.4
C8—C9—H9	123.2 (18)	C32—O3—C33	113.1 (2)
C10—C9—H9	114.6 (18)	C32—O3—K1	110.55 (17)
Co1—C9—H9	115.3 (17)	C33—O3—K1	108.24 (17)
C9—C10—C11	122.9 (3)	O3—C33—C34	107.5 (3)
C9—C10—Co1	68.42 (18)	O3—C33—H33A	110.2
C11—C10—Co1	68.56 (19)	C34—C33—H33A	110.2
C9—C10—H10	120.6 (18)	O3—C33—H33B	110.2
C11—C10—H10	114.5 (17)	C34—C33—H33B	110.2
Co1—C10—H10	125.2 (17)	H33A—C33—H33B	108.5
C12—C11—C10	126.0 (3)	O4—C34—C33	108.7 (3)
C12—C11—Co1	70.29 (18)	O4—C34—H34A	109.9
C10—C11—Co1	71.00 (19)	C33—C34—H34A	109.9
C12—C11—H11	118 (2)	O4—C34—H34B	109.9
C10—C11—H11	115 (2)	C33—C34—H34B	109.9
Co1—C11—H11	124 (2)	H34A—C34—H34B	108.3
C11—C12—C13	128.0 (3)	C34—O4—C35	112.5 (3)
C11—C12—Co1	69.12 (18)	C34—O4—K1	117.73 (18)
C13—C12—Co1	109.1 (2)	C35—O4—K1	116.62 (18)
C11—C12—H12	114 (2)	O4—C35—C36	108.9 (3)
C13—C12—H12	110 (2)	O4—C35—H35A	109.9
Co1—C12—H12	121.0 (19)	C36—C35—H35A	109.9
C12—C13—C14	110.9 (3)	O4—C35—H35B	109.9
C12—C13—H13A	113.4 (18)	C36—C35—H35B	109.9
C14—C13—H13A	110.0 (17)	H35A—C35—H35B	108.3
C12—C13—H13B	110 (2)	O5—C36—C35	109.8 (3)
C14—C13—H13B	111 (2)	O5—C36—H36A	109.7
H13A—C13—H13B	102 (3)	C35—C36—H36A	109.7
C13—C14—C8	111.8 (3)	O5—C36—H36B	109.7
C13—C14—H14A	110.7 (17)	C35—C36—H36B	109.7
C8—C14—H14A	106.4 (17)	H36A—C36—H36B	108.2
C13—C14—H14B	111.2 (17)	C36—O5—C37	112.6 (3)
C8—C14—H14B	110.7 (17)	C36—O5—K1	108.31 (17)
H14A—C14—H14B	106 (2)	C37—O5—K1	106.98 (18)
C22'—Co2—C16	156.4 (7)	O5—C37—C38	108.8 (3)
C22'—Co2—C24'	80.5 (9)	O5—C37—H37A	109.9
C16—Co2—C24'	120.1 (10)	C38—C37—H37A	109.9
C22'—Co2—C15	120.2 (8)	O5—C37—H37B	109.9
C16—Co2—C15	42.17 (16)	C38—C37—H37B	109.9
C24'—Co2—C15	157.8 (8)	H37A—C37—H37B	108.3
C16—Co2—C22	107.4 (4)	O6—C38—C37	108.0 (3)
C15—Co2—C22	120.0 (4)	O6—C38—H38A	110.1
C16—Co2—C24	144.9 (4)	C37—C38—H38A	110.1
C15—Co2—C24	162.9 (4)	O6—C38—H38B	110.1
C22—Co2—C24	75.5 (5)	C37—C38—H38B	110.1
C22'—Co2—C23'	42.2 (6)	H38A—C38—H38B	108.4
C16—Co2—C23'	149.7 (7)	C38—O6—C39	112.7 (2)
C24'—Co2—C23'	41.7 (6)	C38—O6—K1	120.47 (18)
C15—Co2—C23'	160.5 (8)	C39—O6—K1	117.74 (17)
C22'—Co2—C25'	100.1 (10)	O6—C39—C40	107.9 (3)
C16—Co2—C25'	103.2 (10)	O6—C39—H39A	110.1
C24'—Co2—C25'	41.7 (6)	C40—C39—H39A	110.1
C15—Co2—C25'	120.5 (9)	O6—C39—H39B	110.1
C23'—Co2—C25'	76.3 (12)	C40—C39—H39B	110.1
C16—Co2—C23	122.3 (4)	H39A—C39—H39B	108.4
C15—Co2—C23	156.9 (3)	O1—C40—C39	108.9 (3)
C22—Co2—C23	40.3 (3)	O1—C40—H40A	109.9
C24—Co2—C23	40.2 (3)	C39—C40—H40A	109.9
C16—Co2—C25	158.6 (2)	O1—C40—H40B	109.9
C15—Co2—C25	126.2 (3)	C39—C40—H40B	109.9
C22—Co2—C25	93.9 (4)	H40A—C40—H40B	108.3
C24—Co2—C25	39.9 (2)	O7—K2—O9	117.72 (7)
C23—Co2—C25	74.1 (3)	O7—K2—O11	117.43 (7)
C16—Co2—C26	143.25 (19)	O9—K2—O11	116.98 (7)
C15—Co2—C26	102.01 (18)	O7—K2—O8	58.82 (6)
C22—Co2—C26	81.7 (3)	O9—K2—O8	58.94 (6)
C24—Co2—C26	71.6 (3)	O11—K2—O8	150.02 (7)
C23—Co2—C26	88.1 (3)	O7—K2—O10	153.45 (7)
C25—Co2—C26	39.13 (19)	O9—K2—O10	58.17 (6)
C22'—Co2—C26'	86.5 (5)	O11—K2—O10	58.82 (6)
C16—Co2—C26'	109.3 (3)	O8—K2—O10	109.87 (7)
C24'—Co2—C26'	73.6 (8)	O7—K2—O12	58.92 (6)
C15—Co2—C26'	98.3 (3)	O9—K2—O12	157.17 (7)
C23'—Co2—C26'	89.6 (9)	O11—K2—O12	58.77 (6)
C25'—Co2—C26'	39.2 (4)	O8—K2—O12	112.15 (7)
C16—C15—C21	125.7 (3)	O10—K2—O12	113.44 (7)
C16—C15—Co2	65.6 (2)	O7—K2—C20	126.27 (8)
C21—C15—Co2	106.4 (3)	O9—K2—C20	88.19 (10)
C16—C15—K2	72.9 (2)	O11—K2—C20	82.78 (8)
C21—C15—K2	73.3 (2)	O8—K2—C20	124.69 (9)
Co2—C15—K2	127.35 (15)	O10—K2—C20	80.25 (8)
C16—C15—H15	118 (2)	O12—K2—C20	112.20 (9)
C21—C15—H15	112 (2)	O7—K2—C19	109.48 (9)
Co2—C15—H15	120 (2)	O9—K2—C19	113.56 (9)
K2—C15—H15	107 (2)	O11—K2—C19	72.73 (8)
C17—C16—C15	123.2 (3)	O8—K2—C19	137.22 (8)
C17—C16—Co2	80.5 (2)	O10—K2—C19	94.75 (8)
C15—C16—Co2	72.2 (2)	O12—K2—C19	87.36 (8)
C17—C16—K2	86.5 (2)	C20—K2—C19	25.38 (10)
C15—C16—K2	83.5 (2)	O7—K2—C16	72.91 (8)
Co2—C16—K2	139.18 (16)	O9—K2—C16	94.65 (9)
C17—C16—H16	115 (2)	O11—K2—C16	128.96 (8)
C15—C16—H16	122 (2)	O8—K2—C16	80.16 (8)
Co2—C16—H16	118 (2)	O10—K2—C16	131.61 (8)
K2—C16—H16	102 (2)	O12—K2—C16	104.74 (8)
C18—C17—C16	128.3 (4)	C20—K2—C16	57.88 (9)
C18—C17—Co2	111.9 (3)	C19—K2—C16	57.69 (9)
C16—C17—Co2	59.95 (19)	O7—K2—C21	117.55 (9)
C18—C17—H17	111.9 (18)	O9—K2—C21	72.39 (9)
C16—C17—H17	116.8 (18)	O11—K2—C21	105.88 (9)
Co2—C17—H17	116.1 (18)	O8—K2—C21	100.79 (9)
C19—C18—C17	130.6 (4)	O10—K2—C21	87.24 (9)
C19—C18—K2	69.9 (2)	O12—K2—C21	130.20 (9)
C17—C18—K2	84.6 (2)	C20—K2—C21	23.92 (10)
C19—C18—H18	115 (2)	C19—K2—C21	44.53 (10)
C17—C18—H18	114 (2)	C16—K2—C21	44.71 (10)
K2—C18—H18	123 (2)	O7—K2—C18	86.21 (8)
C18—C19—C20	126.9 (4)	O9—K2—C18	125.87 (8)
C18—C19—K2	86.7 (2)	O11—K2—C18	85.43 (8)
C20—C19—K2	77.1 (2)	O8—K2—C18	122.02 (8)
C18—C19—H19	116.7 (19)	O10—K2—C18	118.10 (8)
C20—C19—H19	116.1 (19)	O12—K2—C18	76.96 (8)
K2—C19—H19	112.7 (18)	C20—K2—C18	43.81 (10)
C21—C20—C19	128.0 (4)	C19—K2—C18	23.42 (9)
C21—C20—K2	85.3 (2)	C16—K2—C18	43.92 (9)
C19—C20—K2	77.5 (2)	C21—K2—C18	53.75 (10)
C21—C20—H20	114 (2)	O7—K2—C15	94.16 (8)
C19—C20—H20	117 (2)	O9—K2—C15	75.37 (8)
K2—C20—H20	116 (2)	O11—K2—C15	127.23 (8)
C20—C21—C15	130.0 (4)	O8—K2—C15	82.05 (8)
C20—C21—K2	70.8 (2)	O10—K2—C15	108.57 (8)
C15—C21—K2	82.2 (2)	O12—K2—C15	126.18 (8)
C20—C21—H21	119 (3)	C20—K2—C15	44.99 (9)
C15—C21—H21	111 (3)	C19—K2—C15	56.64 (9)
K2—C21—H21	122 (3)	C16—K2—C15	23.68 (9)
C23—C22—C28	127.2 (10)	C21—K2—C15	24.58 (10)
C23—C22—Co2	71.6 (7)	C18—K2—C15	53.83 (9)
C28—C22—Co2	110.7 (6)	C41—O7—C52	112.1 (3)
C23—C22—H22	113.2	C41—O7—K2	118.45 (18)
C28—C22—H22	113.2	C52—O7—K2	118.42 (19)
Co2—C22—H22	113.2	O7—C41—C42	108.8 (3)
C24—C23—C22	123.1 (8)	O7—C41—H41A	109.9
C24—C23—Co2	68.2 (7)	C42—C41—H41A	109.9
C22—C23—Co2	68.1 (7)	O7—C41—H41B	109.9
C24—C23—H23	118.4	C42—C41—H41B	109.9
C22—C23—H23	118.4	H41A—C41—H41B	108.3
Co2—C23—H23	141.4	O8—C42—C41	109.3 (3)
C25—C24—C23	125.8 (8)	O8—C42—H42A	109.8
C25—C24—Co2	72.5 (5)	C41—C42—H42A	109.8
C23—C24—Co2	71.6 (7)	O8—C42—H42B	109.8
C25—C24—H24	117.1	C41—C42—H42B	109.8
C23—C24—H24	117.1	H42A—C42—H42B	108.3
Co2—C24—H24	132.5	C42—O8—C43	112.6 (3)
C24—C25—C26	119.1 (6)	C42—O8—K2	112.13 (18)
C24—C25—Co2	67.5 (6)	C43—O8—K2	109.72 (18)
C26—C25—Co2	71.9 (4)	O8—C43—C44	108.8 (3)
C24—C25—H25	120.5	O8—C43—H43A	109.9
C26—C25—H25	120.5	C44—C43—H43A	109.9
Co2—C25—H25	133.2	O8—C43—H43B	109.9
C25—C26—C27	118.5 (6)	C44—C43—H43B	109.9
C25—C26—Co2	69.0 (4)	H43A—C43—H43B	108.3
C27—C26—Co2	111.2 (4)	O9—C44—C43	108.1 (3)
C25—C26—H26	116.4	O9—C44—H44A	110.1
C27—C26—H26	116.4	C43—C44—H44A	110.1
Co2—C26—H26	116.4	O9—C44—H44B	110.1
C26—C27—C28	111.1 (4)	C43—C44—H44B	110.1
C26—C27—H27A	109.4	H44A—C44—H44B	108.4
C28—C27—H27A	109.4	C45—O9—C44	112.8 (3)
C26—C27—H27B	109.4	C45—O9—K2	118.99 (18)
C28—C27—H27B	109.4	C44—O9—K2	117.67 (18)
H27A—C27—H27B	108.0	O9—C45—C46	108.8 (3)
C22—C28—C27	110.7 (5)	O9—C45—H45A	109.9
C22—C28—H28A	109.5	C46—C45—H45A	109.9
C27—C28—H28A	109.5	O9—C45—H45B	109.9
C22—C28—H28B	109.5	C46—C45—H45B	109.9
C27—C28—H28B	109.5	H45A—C45—H45B	108.3
H28A—C28—H28B	108.1	O10—C46—C45	108.4 (3)
C23'—C22'—C28'	122.7 (15)	O10—C46—H46A	110.0
C23'—C22'—Co2	77.1 (15)	C45—C46—H46A	110.0
C28'—C22'—Co2	110.6 (9)	O10—C46—H46B	110.0
C23'—C22'—H22'	113.6	C45—C46—H46B	110.0
C28'—C22'—H22'	113.6	H46A—C46—H46B	108.4
Co2—C22'—H22'	113.6	C47—O10—C46	113.4 (3)
C22'—C23'—C24'	118.0 (16)	C47—O10—K2	112.57 (18)
C22'—C23'—Co2	60.7 (12)	C46—O10—K2	108.41 (18)
C24'—C23'—Co2	64.1 (16)	O10—C47—C48	108.3 (3)
C22'—C23'—H23'	121.0	O10—C47—H47A	110.0
C24'—C23'—H23'	121.0	C48—C47—H47A	110.0
Co2—C23'—H23'	154.0	O10—C47—H47B	110.0
C23'—C24'—C25'	126.8 (17)	C48—C47—H47B	110.0
C23'—C24'—Co2	74.2 (16)	H47A—C47—H47B	108.4
C25'—C24'—Co2	74.2 (18)	O11—C48—C47	108.4 (3)
C23'—C24'—H24'	116.6	O11—C48—H48A	110.0
C25'—C24'—H24'	116.6	C47—C48—H48A	110.0
Co2—C24'—H24'	127.4	O11—C48—H48B	110.0
C26'—C25'—C24'	119.3 (12)	C47—C48—H48B	110.0
C26'—C25'—Co2	74.1 (13)	H48A—C48—H48B	108.4
C24'—C25'—Co2	64.1 (16)	C48—O11—C49	112.8 (3)
C26'—C25'—H25'	120.3	C48—O11—K2	115.52 (18)
C24'—C25'—H25'	120.3	C49—O11—K2	117.40 (18)
Co2—C25'—H25'	134.7	O11—C49—C50	108.8 (3)
C25'—C26'—C27'	120.4 (14)	O11—C49—H49A	109.9
C25'—C26'—Co2	66.7 (15)	C50—C49—H49A	109.9
C27'—C26'—Co2	105.7 (6)	O11—C49—H49B	109.9
C25'—C26'—H26C	117.4	C50—C49—H49B	109.9
C27'—C26'—H26C	117.4	H49A—C49—H49B	108.3
Co2—C26'—H26C	117.4	O12—C50—C49	108.3 (3)
C26'—C27'—C28'	111.0 (9)	O12—C50—H50A	110.0
C26'—C27'—H27C	109.4	C49—C50—H50A	110.0
C28'—C27'—H27C	109.4	O12—C50—H50B	110.0
C26'—C27'—H27D	109.4	C49—C50—H50B	110.0
C28'—C27'—H27D	109.4	H50A—C50—H50B	108.4
H27C—C27'—H27D	108.0	C51—O12—C50	113.4 (2)
C22'—C28'—C27'	110.8 (9)	C51—O12—K2	106.40 (18)
C22'—C28'—H28C	109.5	C50—O12—K2	108.45 (18)
C27'—C28'—H28C	109.5	O12—C51—C52	108.0 (3)
C22'—C28'—H28D	109.5	O12—C51—H51A	110.1
C27'—C28'—H28D	109.5	C52—C51—H51A	110.1
H28C—C28'—H28D	108.1	O12—C51—H51B	110.1
O4—K1—O6	117.70 (7)	C52—C51—H51B	110.1
O4—K1—O2	116.76 (7)	H51A—C51—H51B	108.4
O6—K1—O2	118.44 (7)	O7—C52—C51	108.6 (3)
O4—K1—O1	153.61 (7)	O7—C52—H52A	110.0
O6—K1—O1	59.43 (6)	C51—C52—H52A	110.0
O2—K1—O1	59.01 (6)	O7—C52—H52B	110.0
O4—K1—O3	59.05 (7)	C51—C52—H52B	110.0
O6—K1—O3	156.35 (7)	H52A—C52—H52B	108.3
O2—K1—O3	57.77 (6)		
			
C7—C1—C2—C3	35.8 (6)	C23—C22—C28—C27	−39.7 (14)
Co1—C1—C2—C3	−64.5 (3)	Co2—C22—C28—C27	42.5 (9)
K1—C1—C2—C3	84.6 (3)	C26—C27—C28—C22	−36.4 (9)
C7—C1—C2—Co1	100.3 (4)	C16—Co2—C22'—C23'	133.2 (13)
K1—C1—C2—Co1	149.06 (12)	C24'—Co2—C22'—C23'	−19.4 (15)
C7—C1—C2—K1	−48.8 (4)	C15—Co2—C22'—C23'	169.2 (13)
Co1—C1—C2—K1	−149.06 (12)	C25'—Co2—C22'—C23'	−56.4 (14)
C1—C2—C3—C4	−30.0 (5)	C26'—Co2—C22'—C23'	−93.3 (13)
Co1—C2—C3—C4	−91.3 (3)	C16—Co2—C22'—C28'	−106.4 (12)
K1—C2—C3—C4	51.1 (4)	C24'—Co2—C22'—C28'	101.0 (13)
C1—C2—C3—Co1	61.3 (3)	C15—Co2—C22'—C28'	−70.5 (14)
K1—C2—C3—Co1	142.33 (15)	C23'—Co2—C22'—C28'	120.4 (15)
C2—C3—C4—C5	5.5 (6)	C25'—Co2—C22'—C28'	64.0 (13)
Co1—C3—C4—C5	−62.0 (5)	C26'—Co2—C22'—C28'	27.0 (13)
C2—C3—C4—K1	−47.6 (3)	C28'—C22'—C23'—C24'	−76 (4)
Co1—C3—C4—K1	−115.13 (16)	Co2—C22'—C23'—C24'	30 (3)
C3—C4—C5—C6	9.7 (7)	C28'—C22'—C23'—Co2	−106.4 (14)
K1—C4—C5—C6	66.2 (4)	C22'—C23'—C24'—C25'	27 (6)
C3—C4—C5—K1	−56.5 (4)	Co2—C23'—C24'—C25'	56 (4)
C4—C5—C6—C7	−0.9 (6)	C22'—C23'—C24'—Co2	−29 (3)
K1—C5—C6—C7	71.3 (4)	C23'—C24'—C25'—C26'	−4 (6)
C4—C5—C6—K1	−72.2 (4)	Co2—C24'—C25'—C26'	52 (3)
C5—C6—C7—C1	−3.8 (7)	C23'—C24'—C25'—Co2	−56 (4)
K1—C6—C7—C1	68.4 (4)	C24'—C25'—C26'—C27'	48 (4)
C5—C6—C7—K1	−72.1 (4)	Co2—C25'—C26'—C27'	95.1 (14)
C2—C1—C7—C6	−16.2 (7)	C24'—C25'—C26'—Co2	−47 (3)
Co1—C1—C7—C6	59.6 (5)	C25'—C26'—C27'—C28'	−87.4 (19)
K1—C1—C7—C6	−65.2 (4)	Co2—C26'—C27'—C28'	−16 (2)
C2—C1—C7—K1	49.0 (4)	C23'—C22'—C28'—C27'	44 (2)
Co1—C1—C7—K1	124.8 (2)	Co2—C22'—C28'—C27'	−43 (2)
C14—C8—C9—C10	46.8 (4)	C26'—C27'—C28'—C22'	38 (2)
Co1—C8—C9—C10	−55.3 (3)	C40—O1—C29—C30	176.3 (3)
C14—C8—C9—Co1	102.1 (3)	K1—O1—C29—C30	−60.1 (3)
C8—C9—C10—C11	12.5 (5)	O1—C29—C30—O2	66.3 (3)
Co1—C9—C10—C11	−43.7 (3)	C29—C30—O2—C31	176.0 (3)
C8—C9—C10—Co1	56.1 (3)	C29—C30—O2—K1	−38.9 (3)
C9—C10—C11—C12	−2.7 (5)	C30—O2—C31—C32	178.7 (3)
Co1—C10—C11—C12	−46.3 (3)	K1—O2—C31—C32	34.5 (3)
C9—C10—C11—Co1	43.6 (3)	O2—C31—C32—O3	−64.0 (3)
C10—C11—C12—C13	−51.8 (5)	C31—C32—O3—C33	−177.0 (2)
Co1—C11—C12—C13	−98.4 (3)	C31—C32—O3—K1	61.4 (3)
C10—C11—C12—Co1	46.6 (3)	C32—O3—C33—C34	175.3 (3)
C11—C12—C13—C14	36.0 (5)	K1—O3—C33—C34	−61.9 (3)
Co1—C12—C13—C14	−42.0 (3)	O3—C33—C34—O4	69.3 (3)
C12—C13—C14—C8	35.9 (4)	C33—C34—O4—C35	−179.6 (3)
C9—C8—C14—C13	−88.9 (4)	C33—C34—O4—K1	−39.5 (3)
Co1—C8—C14—C13	−13.0 (3)	C34—O4—C35—C36	−178.3 (3)
C21—C15—C16—C17	−27.8 (6)	K1—O4—C35—C36	41.2 (4)
Co2—C15—C16—C17	65.4 (3)	O4—C35—C36—O5	−69.5 (4)
K2—C15—C16—C17	−81.4 (3)	C35—C36—O5—C37	177.2 (3)
C21—C15—C16—Co2	−93.2 (4)	C35—C36—O5—K1	59.1 (3)
K2—C15—C16—Co2	−146.80 (12)	C36—O5—C37—C38	175.7 (3)
C21—C15—C16—K2	53.6 (3)	K1—O5—C37—C38	−65.5 (3)
Co2—C15—C16—K2	146.80 (12)	O5—C37—C38—O6	65.2 (3)
C15—C16—C17—C18	33.9 (6)	C37—C38—O6—C39	−176.5 (3)
Co2—C16—C17—C18	95.2 (4)	C37—C38—O6—K1	−30.2 (3)
K2—C16—C17—C18	−45.9 (4)	C38—O6—C39—C40	−174.4 (3)
C15—C16—C17—Co2	−61.4 (3)	K1—O6—C39—C40	38.2 (3)
K2—C16—C17—Co2	−141.17 (13)	C29—O1—C40—C39	−175.0 (3)
C16—C17—C18—C19	−12.3 (7)	K1—O1—C40—C39	60.8 (3)
Co2—C17—C18—C19	55.9 (5)	O6—C39—C40—O1	−66.8 (3)
C16—C17—C18—K2	45.2 (4)	C52—O7—C41—C42	−174.6 (3)
Co2—C17—C18—K2	113.49 (19)	K2—O7—C41—C42	41.9 (3)
C17—C18—C19—C20	−7.6 (6)	O7—C41—C42—O8	−64.5 (4)
K2—C18—C19—C20	−71.2 (3)	C41—C42—O8—C43	179.1 (3)
C17—C18—C19—K2	63.5 (4)	C41—C42—O8—K2	54.8 (3)
C18—C19—C20—C21	2.0 (6)	C42—O8—C43—C44	173.0 (3)
K2—C19—C20—C21	−73.8 (4)	K2—O8—C43—C44	−61.4 (3)
C18—C19—C20—K2	75.8 (4)	O8—C43—C44—O9	67.3 (3)
C19—C20—C21—C15	9.0 (7)	C43—C44—O9—C45	177.5 (3)
K2—C20—C21—C15	−61.2 (4)	C43—C44—O9—K2	−38.1 (3)
C19—C20—C21—K2	70.2 (4)	C44—O9—C45—C46	173.8 (3)
C16—C15—C21—C20	3.2 (7)	K2—O9—C45—C46	29.9 (3)
Co2—C15—C21—C20	−68.2 (5)	O9—C45—C46—O10	−64.5 (4)
K2—C15—C21—C20	56.6 (4)	C45—C46—O10—C47	−168.7 (3)
C16—C15—C21—K2	−53.5 (3)	C45—C46—O10—K2	65.5 (3)
Co2—C15—C21—K2	−124.89 (17)	C46—O10—C47—C48	−178.3 (3)
C28—C22—C23—C24	60 (2)	K2—O10—C47—C48	−54.8 (3)
Co2—C22—C23—C24	−42.2 (14)	O10—C47—C48—O11	69.0 (3)
C28—C22—C23—Co2	102.4 (11)	C47—C48—O11—C49	173.5 (3)
C22—C23—C24—C25	−9 (3)	C47—C48—O11—K2	−47.5 (3)
Co2—C23—C24—C25	−51.0 (13)	C48—O11—C49—C50	174.7 (3)
C22—C23—C24—Co2	42.2 (14)	K2—O11—C49—C50	36.6 (3)
C23—C24—C25—C26	−1.4 (19)	O11—C49—C50—O12	−68.3 (3)
Co2—C24—C25—C26	−52.0 (8)	C49—C50—O12—C51	−178.6 (3)
C23—C24—C25—Co2	50.6 (13)	C49—C50—O12—K2	63.4 (3)
C24—C25—C26—C27	−53.4 (11)	C50—O12—C51—C52	174.5 (3)
Co2—C25—C26—C27	−103.4 (5)	K2—O12—C51—C52	−66.4 (3)
C24—C25—C26—Co2	50.0 (9)	C41—O7—C52—C51	−175.3 (3)
C25—C26—C27—C28	91.0 (9)	K2—O7—C52—C51	−31.7 (3)
Co2—C26—C27—C28	14.1 (9)	O12—C51—C52—O7	67.4 (3)
